# Development of Left Ventricular Longitudinal Speckle Tracking Echocardiography in Very Low Birth Weight Infants with and without Bronchopulmonary Dysplasia during the Neonatal Period

**DOI:** 10.1371/journal.pone.0106504

**Published:** 2014-09-03

**Authors:** Christoph Czernik, Stefanie Rhode, Sven Helfer, Gerd Schmalisch, Christoph Bührer, Lothar Schmitz

**Affiliations:** 1 Department of Neonatology, Charité University Medical Center, Berlin, Germany; 2 Department of Pediatric Cardiology, Charité University Medical Center, Berlin, Germany; Hôpital Robert Debré, France

## Abstract

**Objectives:**

In preterm infants, postnatal myocardial adaptation may be complicated by bronchopulmonary dysplasia (BPD). We aimed to describe the development of left ventricular function by serial 2D, Doppler, and speckle tracking echocardiography (2D-STE) in infants with and without BPD during the neonatal period and compare these to anthropometric and conventional hemodynamic parameters.

**Study Design:**

Prospective echocardiography on day of life (DOL) 1, 7, 14, and 28 in 119 preterm infants <1500 g birth weight of whom 36 developed BPD (need for oxygen supplementation at 36 weeks gestational age). Non-BPD and BPD infants differed significantly in median (IQR) gestational age (25.5(24–26.5) weeks vs. 29(27–30) weeks, p<0.001) and birth weight (661(552–871) g vs. 1100(890–1290) g, p<0.001).

**Results:**

The intra- and inter-observer variability of the 2D-STE parameters measured did not depend on time of measurement, although there were significant differences in the reproducibility of the parameters. Low intra- and inter-observer variability was seen for longitudinal systolic strain and strain rate mid septum with a median CV (coefficient of variation) of <4.6%. Much higher CVs (>10%) were seen for the apical segment. While anthropometric parameters show rapid development during the first 4 weeks of life, the speckle tracking parameters did not differ statistically significantly during the neonatal period. Infants with and without BPD differed significantly (p<0.001) in the development of anthropometric parameters, conventional hemodynamic parameters except for heart rate, and 2D-STE parameters: global longitudinal systolic strain rate (GLSSR) and longitudinal systolic strain for the mid left wall (LSSR). The largest differences were seen at DOL 1 and 7 in GLSSR (p<0.001) and in LSSR (p<0.01).

**Conclusions:**

Reproducible 2D-STE measurements are possible in preterm infants <1500 g. Cardiac deformation reveals early (DOL 1 and 7) ventricular changes (GLSSR and LSSR) in very low birth weight infants who develop BPD.

## Introduction

After birth, newborn life is dominated by a process of adaption. In order to survive deprived of the maternal supply of oxygen and metabolites, the infant’s organism has to develop significantly in the first hours and days of life [1], [2]. One of the most important developments here is the transition from fetal to postnatal circulation. For preterm and particularly for very low birth weight (VLBW) infants (birth weight<1500 g), this transition is especially difficult. While the pathophysiologic processes that lead to bronchopulmonary dysplasia (BPD) and patent ductus arteriosus (PDA) are still incompletely understood, they are closely linked to the immaturity of the cardiorespiratory system in this population [3], [4]. BPD development is based on the immaturity of the lung tissue in combination with oxidative stress [5]. Increased right ventricular afterload and pulmonary hypertension (PH) are common complications of BPD, and recent studies have increased awareness that PH worsens the clinical course, morbidity and mortality of BPD [6].

In order to better monitor the cardiopulmonary development of these patients, new diagnostic tools are always needed and emerging [7]. One of these new tools is two-dimensional speckle tracking echocardiography (2D-STE) based strain and strain rate measurements. 2D-STE is based on classic B-mode imaging [8]–[10]. Speckles are groups of pixels that represent the acoustic backscatter of the ultrasonic beam. These form the characteristic B-mode image. Special software algorithms can track these patterns and calculate velocities and deformation. As opposed to measurements taken using tissue Doppler imaging (TDI), this allows for relative angle independency within the 2D sector, and thus two-dimensional strain and strain rate measurements.

2D-STE based deformation imaging has shown promising results in various clinical studies and its reliability and repeatability has been demonstrated in adult and pediatric populations [11]–[17]. Nestaas et al. were able to show that TDI derived strain was more sensitive than fractional shortening (FS) in detecting myocardial dysfunctions in asphyxiated newborns during and after whole-body therapeutic hypothermia [18], [19], and we were able to show that hypothermia also affects 2D-STE based strain rate [20]. In their study, Hardegree et al. note that the right (RV) and left ventricles (LV) do not function in isolation in a patient with PH and found not only a reduction in RV systolic strain, but also reduced LV systolic strain [21].

These results are encouraging and indicate that 2D-STE might also deliver new insights into the cardiac function of very low birth weight infants. In contrast to anthropometric and conventional hemodynamic parameters, very little is known about the development of 2D-STE parameters during the neonatal period, especially in patients who develop BPD. We hypothesized that there are significant differences in the development of the speckle tracking parameters between infants who subsequently did and did not develop BPD. Therefore, the goal of this study was to assess the applicability and reproducibility of the speckle tracking parameters in VLBW infants and the development of 2D-STE based strain and strain rate in the first 28 days of life compared to hemodynamic and anthropometric parameters.

## Patients and Methods

### Study population

The study was conducted prospectively at our perinatal center from September 2008 to January 2011. During the study period, we enrolled 119 of the 167 VLBW infants who were admitted to our neonatal intensive care unit. 48 infants were excluded from our study. Of these, 5 had congenital heart malformations, and for 7 no parental consent was given. 18 infants died within 48 h after birth and 18 in the first month of life, so they did not have the full set of four sequential echocardiograms and were therefore later excluded from the study. All infants received standard intensive care, no infant required inotropes or vasopressors, and they were examined without sedation. The study protocol was approved by the local institutional review board (Ethikkommission der Charité. # EA2/072/08), and written informed parental consent was obtained.

Weight and length at birth and at 1, 7, 14 and 28 days of life were documented. Bronchopulmonary dysplasia, defined as a requirement for supplemental oxygen at 36 weeks gestational age, was recorded as morbidity. We also recorded when hemodynamically significant PDA (hsPDA) treatment (either medical or surgical) was required using the following previously established criteria: PDA with left-to-right-shunt, narrowest diameter >2 mm in addition to the need for invasive ventilatory support [22].

### Blood pressure measurement

Noninvasive systolic, diastolic and mean arterial blood pressure were assessed using an IntelliVue MMSX2 (Philips) monitor and recorded at the same time as echocardiographic measurement. Philips infant BP cuffs were used (sizes 1 to 3). The smallest cuff size that covered at least two-thirds of the right upper arm or encompassed the entire arm was selected.

### Conventional echocardiographic measurements

All patients were examined using the same 7S transducer interfaced with a Vingmed System Vivid 7 Dimension’06 (GE Vingmed, Horten, Norway). Transthoracic imaging was performed on the neonates at the ages of 1, 7, 14 and 28 days in a supine position without sedation. All scans were performed by the same experienced operator.

M-mode tracings of the left ventricular cavity were recorded from the parasternal long-axis view with a single beam directed at the level of the tips of the mitral leaflets. We used LV fractional shortening (FS) as a measure of systolic left ventricular function. FS was defined as the difference in left ventricular end-diastolic diameter (LVEDD) and left ventricular end-systolic diameter (LVESD) divided by LVEDD and then multiplied by 100.

Left ventricular output (LVO) was calculated as the total time velocity integral of mitral inflow multiplied by mitral valve (MV) area and heart rate, then divided by body weight. The diameter of the MV annulus was measured using the end-diastolic hinge points extracted from the apical four-chamber view frames. The MV area was calculated from the MV diameter in diastole assuming circularity of the left ventricle. The total time velocity integral of mitral inflow was measured using pulsed wave Doppler from the same apical four-chamber view. The measurement procedure is standard throughout our department and a detailed description has already been published [23]. At least three consecutive cardiac cycles were recorded for all parameters and the mean values of each parameter were used for further analysis. Offline analysis of the data was performed using dedicated software (EchoPac PC SW 108.1.9, GE Vingmed, Horten, Norway).

### 2D speckle tracking echocardiography

We acquired LV four-chamber views using a median frame rate of 115 frames per second. Five consecutive cardiac cycles were acquired for each plane and digitally stored on a hard drive for offline analysis. Image analysis was performed offline on a PC workstation using custom analysis software. Strain quantification was performed using commercially available software (EchoPAC PC version 108.1.9, GE Vingmed, Horten, Norway).

**Figure 1:**
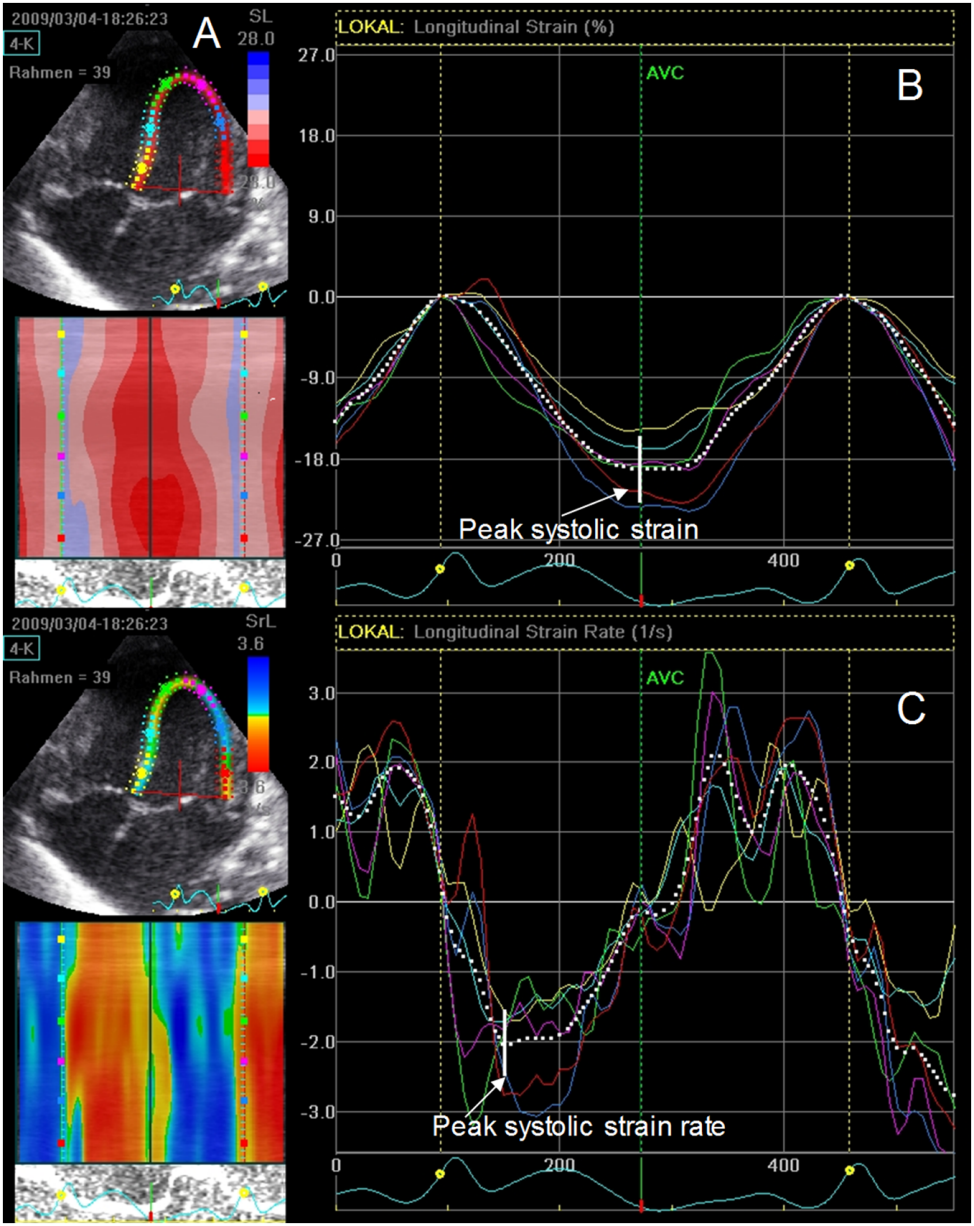
Measurement of peak systolic strain and strain rate. (A) 2D apical four-chamber view of the left ventricle with region of interest (in color with each color representing one of six segments) encompassing endo- and epicardium for 2D strain and strain rate analysis. (B) Global longitudinal strain (white dotted line) was calculated by averaging measurements of six segments in the apical four-chamber view. (C) Global longitudinal strain rate (white dotted line) was calculated by averaging measurements of six segments in the apical four-chamber view.

In all patients, the left ventricle endocardial border in the end-systolic frame was manually traced during the automatically selected next to last cycle. Based on this line, the software automatically created a region of interest that included the mid layer of the ventricular wall. Linear drift compensation was applied. In the present study, peak systolic longitudinal strain and strain rate were assessed in the six LV regions in the apical four-chamber view, and their average values were displayed by the device expressed as global longitudinal strain and strain rate (Figure 1). Strain and strain rate analyses of all included patients were performed by the same observer who was blind to the clinical condition of the infants.

### Statistical analysis

Patient characteristics were described as rates (%) or median and interquartile ranges (IQR). To describe the intra-observer variability of the speckle tracking parameters, 20 randomly selected patients were reevaluated by the same investigator (SR) on two separate occasions and the coefficient of variation (CV) calculated for the four measurement points. To describe inter-observer variability, the same procedure was repeated by a second observer (CC). The CVs of the repeated measurements were presented as medians and quartiles and the Friedman test was used to investigate the changes in intra- and inter-observer variability at the four time points. The Kruskal-Wallis test was used to compare the intra- and inter-observer variability between the different speckle tracking parameters. The Intraclass correlation coefficient (absolute agreement) (ICC) of the pooled data was calculated to describe the reliability of the speckle tracking measurements. The anthropometric and cardiological parameters measured and calculated were depicted as median and IQR in the text and in the figures: differences at the 4 measurement time points were investigated using the Friedman test. To investigate differences in the parameter development between infants with and without BPD development or hsPDA, a two-factor ANOVA for repeated measurements with BPD or hsPDA as a between-subject factor was used. If statistically significant differences in the parameter development emerged, median and IQR of these parameters were calculated for both patient groups at the four time points and the Mann-Whitney rank test was used post hoc to investigate the differences. The relationships LVO, FS, GLSSR and LSSR with the heart rate were assessed by Spearman rank order correlation coefficients (*R_s_*). The Bonferroni method was used as an alpha adjustment for multiple comparisons. Statistical analysis was performed using Statgraphics Centurion (Version 16.0, Statpoint Inc., Herndon, Verginia, USA) and MedCalc (Version 9.2.0.2; MedCalc Software, Mariakerke, Belgium) software. A p-value below 0.05 was considered the limit for statistical significance.

## Results

### Subjects

The patient characteristics of the 119 VLBW infants investigated are shown in Table 1. About two thirds of enrolled patients required mechanical ventilation during the first 4 weeks of life. A total of 36 (30%) infants developed BPD and 33 (28%) infants required PDA intervention. 34 of 36 (94%) BPD infants were mechanically ventilated. BPD infants had a significantly lower gestational age (25.5 (24–26.5) weeks vs. 29 (27–30) weeks, p<0.001) and birth weight (661 (552–871) g vs. 1100 (890–1290) g, p<0.001). The median age for ductus closure was between the second and third week of life. 27 of 36 (75%) BPD infants needed a PDA intervention.

**Table 1:** Characteristics of the study population (N = 119) (Data given as median (IQR) or n(%)).

	No (%)	Median (IQR)
Gestational age (weeks)		27 (26.0–29.0)
Birth weight (g)		996 (745–1200)
SGA	37 (31%)	
Male	58 (49%)	
Antenatal steroids for fetal lung maturation	110 (92%)	
Surfactant administration	101 (85%)	
Mechanical ventilation	76 (64%)	
BPD	36 (30%)	
PDA intervention	33 (28%)	
Time of PDA intervention (days)		19 (14–24)
Alive	116 (97%)	

### Reproducibility of the speckle tracking parameters

Table 2 shows intra-observer variability measured by the CV of the speckle tracking parameters. After Bonferroni correction, there were no statistically significant differences in intra-observer variability among the four time points. It was also noted that the CVs of inter-observer variability were not dependent on time of measurement, as shown in Table 3. Therefore the intra- and inter-observer CVs of the four time points were pooled and the ICC calculated (Table 4).

**Table 2:** Intra-observer variability of the speckle tracking parameters measured by the coefficient of variation of repeated measurements at day of life (DOL) 1, 7, 14, and 28.

Parameter	DOL 1	DOL 7	DOL 14	DOL 28	P-value
Global longitudinal peak systolicstrain	4.3%	5.3%	5.2%	5.5%	0.810
	(0.9%–7.4%)	(3.2%–9.3%)	(2.6%–9.9%)	(1.6%–8.9%)	
Global longitudinal peak systolicstrain rate	3.6%	3.9%	6.5%	4.6%	0.842
	(1.1%–11.4%)	(2.2%–9.9%)	(2.5%–12.0%)	(2.6%–8.3%)	
*Longitudinal peak systolic strain*					
Septum					
basal	6.0%	6.1%	5.9%	4.8%	0.354
	(3.9%–8.7%)	(2.3%–8.9%)	(3.8%–10.7%)	(2.5%–7.2%)	
mid	4.3%	3.9%	3.2%	3.4%	0.492
	(2.6%–9.6%)	(2.5%–9.7%)	(2.2%–4.4%)	(1.7%–7.9%)	
apical	10.9%	13.2%	9.2%	9.8%	0.678
	(4.6%–30.3%)	(7.2%–19.5%)	(5.5%–16.4%)	(4.6%–14.6%)	
Left wall					
basal	13.7%	15.4%	10.7%	9.2%	0.526
	(5.6%–23.3%)	(6.4%–35.5%)	(2.5%–26.6%)	(2.9%–29.5%)	
mid	7.5%	11.8%	10.5%	11.3%	0.785
	(2.4%–14.2%)	(7.6%–24.6%)	(4.4%–16.6%)	(5.4%–23.0%)	
apical	16.6%	9.8%	22.3%	11.8%	0.611
	(8.7%–24.7%)	(2.4%–25.9%)	(10.9%–35.2%)	(7.6%–24.6%)	
*Longitudinal peak systolic strain rate*					
Septum					
basal	8.3%	4.5%	6.8%	7.2%	0.392
	(5.2%–14.9%)	(2.9%–8.8%)	(3.2%–8.6%)	(2.1%–9.7%)	
mid	3.3%	4.4%	5.2%	3.1%	0.260
	(1.3%–7.7%)	(2.0%–10.4%)	(2.4%–7.9%)	(2.0%–7.2%)	
apical	7.9%	9.7%	9.0%	9.4%	0.975
	(2.9%–16.5%)	(5.9%–16.4%)	(5.9%–17.7%)	(3.6%–13.7%)	
Left wall					
basal	17.1%	12.2%	5.1%	6.3%	0.026
	(13.7%–23.6%)	(4.5%–23.0%)	(2.4%–15.6%)	(3.4%–13.9%)	
mid	10.5%	10.0%	12.2%	12.9%	0.654
	(5.9%–18.2%)	(4.7%–22.3%)	(5.1%–19.1%)	(6.0%–19.0%)	
apical	10.1%	19.9%	25.1%	15.2%	0.126
	(3.8%–19.4%)	(7.7%–20.0%)	(7.7%–33.1%)	(9.0%–20.8%)	

Presented are median and interquartile range, statistical significance after Bonferroni correction for p<0.0036.

**Table 3:** Inter-observer variability of the speckle tracking parameters measured by the coefficient of variation of two different observers at day of life (DOL) 1, 7, 14, and 28.

Parameter	DOL 1	DOL 7	DOL 14	DOL 28	P-value
Global longitudinal peak systolicstrain	3.8%	4.0%	6.6%	5.2%	0.119
	(1.6%–6.9%)	(1.5%–6.7%)	(2.5%–13.3%)	(3.1%–7.7%)	
Global longitudinal peak systolicstrain rate	3.8%	4.9%	3.4%	4.9%	0.686
	(1.6%–8.8%)	(1.9%–7.1%)	(2.0%–5.4%)	(2.3%–6.6%)	
*Longitudinal peak systolic strain*					
Septum					
basal	8.9%	2.0%	5.8%	8.5%	0.134
	(4.5%–14.3%)	(1.2%–10.4%)	(3.9%–14.6%)	(2.9%–14.5%)	
mid	5.2%	3.4%	4.1%	5.6%	0.648
	(4.5%–14.3%)	(1.9%–8.3%)	(1.9%–6.6%)	(2.0%–9.9%)	
apical	11.4%	5.4%	5.4%	7.8%	0.325
	(6.2%–16.8%)	(2.7%–10.2%)	(3.1%–11.8%)	(2.6%–14.9%)	
Left wall					
basal	6.6%	9.6%	19.4%	11.1%	0.093
	(4.9%–19.7%)	(3.5%–14.3%)	(13.3%–29.8%)	(6.5%–20.5%)	
mid	6.2%	6.5%	14.8%	6.5%	0.134
	(2.8%–13.2%)	(1.5%–17.7%)	(4.9%–24.2%)	(3.3%–12.7%)	
apical	9.0%	9.2%	14.9%	6.7%	0.187
	(3.8%–18.2%)	(5.9%–17.8%)	(10.5%–30.4%)	(2.5%–23.0%)	
*Longitudinal peak systolic strain rate*					
Septum					
basal	8.2%	6.5%	7.5%	4.8%	0.121
	(7.1%–13.7%)	(1.9%–9.0%)	(4.5%–11.9%)	(3.2%–10.4%)	
mid	5.5%	4.4%	3.6%	6.4%	0.860
	(2.9%–7.3%)	(3.1%–6.8%)	(2.1%–7.8%)	(1.9%–11.1%)	
apical	7.9%	9.7%	9.0%	9.4%	0.903
	(2.9%–16.5%)	(5.9%–16.4%	(5.9%–17.7%)	(3.6%–13.7%)	
Left wall					
basal	11.2%	8.9%	10.4%	9.2%	0.418
	(6.8%–16.9%)	(6.4%–10.6%)	(6.8%–14.0%)	(2.2%–15.5%)	
mid	7.9%	9.3%	8.7%	3.8%	0.550
	(4.2%–13.5%)	(4.2%–18.0%)	(3.7%–13.1%)	(1.8%–14.2%)	
apical	8.6%	11.2%	13.5%	9.4%	0.550
	(4.6%–12.1%)	(5.4%–21.8%)	(4.7%–21.1%)	(6.1%–21.1%)	

Presented are median and interquartile range.

**Table 4:** Comparison of intra- and inter-observer variability of the speckle tracking parameters by the coefficient of variation and intraclass-correlation coefficient.

Parameter	Intra-observer variability		Inter-observer variability		P-value1)
	Coefficient ofvariation	Intraclass-correlationcoefficient	Coefficient ofvariation	Intraclass-correlationcoefficient	
Global longitudinal peak systolicstrain	5.1%	0.884	4.7%	0.904	0.861
	(1.8%–9.0%)		(1.7%–7.8%)		
Global longitudinal peak systolicstrain rate	4.5%	0.890	3.9%	0.938	0.195
	(2.2%–9.6%)		(2.2%–6.4%)		
*Longitudinal peak systolic strain*					
Septum					
basal	5.5%	0.913	6.7%	0.914	0.370
	(3.3%–8.5%)		(2.4%–12.3%)		
mid	3.7%	0.911	4.6%	0.918	0.622
	(2.2%–7.8%)		(2.0%–8.8%)		
apical	10.8%	0.832	6.7%	0.829	0.014
	(5.5%–17.1%)		(3.0%–14.1%)		
Left wall					
basal	12.7%	0.845	11.8%	0.866	0.595
	(4.8%–28.1%)		(5.8%–22.0%)		
mid	10.3%	0.846	8.1%	0.900	0.120
	(4.5%–19.7%)		(2.8%–16.4%)		
apical	13.7%	0.835	10.4%	0.832	0.090
	(7.4%–29.1%)		(4.2%–23.4%)		
*Longitudinal peak systolic strain rate*					
Septum					
basal	7.0%	0.868	7.1%	0.866	0.668
	(3.1%–9.9%)		(3.7%–10.6%)		
mid	3.7%	0.890	4.4%		0.513
	(2.0%–8.2%)		(2.4%–8.0%)	0.900	
apical	8.7%	0.897	7.7%	0.903	0.161
	(4.9%–16.4%)		(3.2%–11.6%)		
Left wall					
basal	9.9%	0.786	9.9%	0.758	0.469
	(4.8%–19.7%)		(4.8%–13.9%)		
mid	11.3%	0.814	8.7%	0.824	0.010
	(5.3%–18.7%)		(3.7%–13.1%)		
apical	14.0%	0.832	10.8%	0.864	0.120
	(7.3%–27.5%)		(4.7%–20.6%)		

Presented are median and interquartile range; statistical significance after Bonferroni correction for p<0.0036.

1) Comparison of the coefficient of variation between intra-and inter-observer variability.

As shown in Table 4, there were distinct differences in the CVs between the parameters measured. The best reproducibility with the lowest intra-observer variability was seen with the longitudinal systolic strain (ICC = 0.911) and strain rate (ICC = 0.89) of the mid septum with a median CV = 3.7% for both. Compared to the mid septum, the CVs of the left wall were distinctly higher. The highest intra-observer variability was found in the apical segment.

After Bonferroni correction, the CVs of inter-observer variability did not differ significantly from the intra-observer CVs (Table 4). The lowest inter-observer variabilities and the highest ICC were also seen for the global longitudinal peak systolic strain rate (median CV 3.9%, ICC = 0.938) and longitudinal systolic strain and strain rate of the mid septum (median CV≤4.6%, ICC≥0.9). Much higher variabilities (CV>10%) were seen for the apical segment.

### Anthropometric, hemodynamic and speckle tracking parameters

Table 5 summarizes the results of the anthropometric, conventional haemodynamic and speckle tracking measurements. It was not possible to obtain complete data at all 4 times of measurement for all 119 infants due to a lack of documentation of anthropometric data or suboptimal imaging quality. Table 5 shows the number of infants with complete data sets for each parameter at all four time points. The number of complete data sets of the left wall was lower than for the septum due to the poorer image quality of the left wall.

**Table 5:** Development of anthropometric, conventional hemodynamic and speckle tracking parameters during the neonatal period.

Parameter	N	DOL 1	DOL 7	DOL 14	DOL 28	p-value
*Anthropometric parameters*						
Body weight (g)	117	970	978	1135	1460	**<0.001**
		(749.5–1198)	(758–1214)	(842–1400)	(1035–1792)	
Body length (cm)	114	36	37	37	39.5	**<0.001**
		(33–38)	(34–39)	(34–40)	(36–42)	
*Conventional hemodynamic and echocardiographic parameters*						
Heart rate (beat/min)	96	152	164	164	164	**<0.001**
		(142–159.5)	(155–173)	(153–173)	(152–174)	
Systolic blood pressure (mm Hg)	96	49	57	60	65	**<0.001**
		(42–57)	(50–64)	(53–66)	(57–73.5)	
Diastolic blood pressure (mm Hg)	96	33	35	35	39	**<0.001**
		(27.5–37)	(29–41)	(28–41)	(32–47)	
Mean blood pressure (mm Hg)	96	38	43	42	48	**<0.001**
		(33.5–42.5)	(36–48)	(37.5–48.5)	(42–54)	
Fractional shortening (%)	96	30.6	34.9	34.7	34.9	**<0.001**
		(26.9–35.7)	(29.0–40.1)	(30.4–40.2)	(29.7–38.3)	
LVO (mL/min/kg)	91	311	360	388	550	**<0.001**
		(242–390)	(289–468)	(291–500)	(441–683)	
*Speckle tracking parameters*						
Global longitudinal peak systolic strain (%)	96	−15.05	−15.15	−15.5	−15.05	0.712
		(−15.5–−12.6)	(−16.8–−12.6)	(−17.4–−13.4)	(−17.4–−13.4)	
Global longitudinal peak systolic strain rate (1/sec)	96	−1.4	−1.5	−1.6	−1.6	0.023
		(−1.7–−1.2)	(−1.85–−1.3)	(−1.8–−1.35)	(−1.75–−1.4)	
Longitudinal peak systolic strain (%)						
Septum						
basal	96	−15.7	−15.8	−15.7	−14.6	0.438
		(−17.9–−12.8)	(−17.3–−12.7)	(−17.7–−13.0)	(−17.0–−12.6)	
mid	97	−16.6	−17.0	−17.2	−16.9)	0.552
		(−19.4–−14.7)	(−18.7–−14.2)	(−19.0–−15.5)	(−18.9–−14.8)	
apical	83	−16.3	−16.4	−18.4	−17.9	0.030
		(−19.8–−11.8)	(−20.4–−13.0)	(−22.0–−14.6)	(−21.4–−15.3	
Left wall						
basal	76	−13.7	−12.8	−12.2	−12.6	0.737
		(−18.4–−7.3)	(−17.3–−8.7)	(−18.4–−7.9)	(−16.7–−8.2)	
mid	84	−12.7	−11.6	−13.5	−13.4	0.203
		(−18.0–−7.8)	(−15.1–−8.4)	(−17.5–−10.1)	(−16.3–−10.4)	
apical	65	−12.2	−12.7	−17.4	−16.6	0.202
		(−18.3–−7.2)	(−18.9–−8.9)	(−20.9–−9.3)	(−21.5–−10.5)	
Longitudinal peak systolic strain rate (1/sec)						
Septum						
basal	93	−1.66	−1.75	−1.71	−1.69	0.332
		(−1.95–−1.41)	(−2.03–−1.56)	(−2.05–−1.56)	(−1.95–−1.48)	
mid	97	−1.66	−1.79	−1.83	−1.81	0.043
		(−1.97–−1.45)	(−2.0–−1.62)	(−2.12–−1.58)	(−2.0–−1.6)	
apical	84	−1.78	−1.98	−2.14	−2.18	**0.001**
		(−2.29–−1.44)	(−2.46–−1.58)	(−2.67–−1.77)	(−2.52–−1.72)	
Left wall						
basal	86	−2.07	−2.21	−2.0	−1.99	0.157
		(−2.62–−1.67)	(−2.56–−1.83)	(−2.39–−1.72)	(−2.46–−1.59)	
mid	91	−1.57	−1.72	−1.65	−1.78	0.358
		(−1.95–−1.28)	(−2.0–−1.37)	(−1.95–−1.42)	(−2.07–−1.45)	
apical	79	−1.51	−1.7	−1.96	−1.86	0.041
		(−2.28–−1.13)	(−2.63–−1.26)	(−2.35–−1.31)	(−2.4–−1.46)	

Presented are median and interquartile range in brackets; statistically significant p-values of the Friedman test after Bonferroni correction (p<0.0023) are printed in bold.

N- Number of infants with complete data in the follow up, DOL- day of life, LVO - Left ventricular output.

The results indicate statistically significant differences between the four measurement time points for all parameters except for almost all speckle tracking parameters. Body weight, body length and blood pressure increased continuously during the neonatal period. Heart rate and fractional shortening as well as LVO differed statistically significantly only during the first days of life; on day 7, 14 and 28 these parameters did not show any statistically significant changes.

For the speckle tracking parameters only the longitudinal peak systolic strain (LSS) for the septum apical segment showed statistically significant parameter development after Bonferroni correction of the p-value. However, LSS was only significantly reduced at day 1; on day 7, 14 and 28 LSS did not show statistically significant changes (p = 0.114).

### Parameter development in infants with and without developing BPD or hsPDA

The differences in the development of anthropometric, conventional hemodynamic, and speckle tracking parameters between infants with and without BPD or with and without hsPDA are shown in Table 6. The development of the anthropometric parameters, blood pressure, FS and LVO differed significantly in infants who developed a BPD or hsPDA. Only two speckle tracking parameter were affected by developing BPD but not by hsPDA. After Bonferroni correction, there was no statistically significant interaction between BPD or hsPDA and age.

**Table 6:** Influence of BPD development and hemodynamically patent ductus arteriosus (hsPDA) on the measured parameters during the neonatal period.

Parameter	BPD			hsPDA		
	BPD	DOL	Interaction	hsPDA	DOL	Interaction
*Anthropometric parameters*						
Body weight (g)	**<0.001**	**<0.001**	0.008	**<0.001**	**<0.001**	0.027
Body length (cm)	**<0.001**	**<0.001**	0.011	**<0.001**	**<0.001**	0.037
*Conventional hemodynamic and echocardiographic parameters*						
Heart rate (beat/min)	0.277	**<0.001**	0.182	0.219	**<0.001**	0.004
Blood pressure (mm Hg)						
systolic	**<0.001**	**<0.001**	0.096	**<0.001**	**<0.001**	0.083
diastolic	**0.001**	**<0.001**	0.182	**<0.001**	**<0.001**	0.152
mean	**<0.001**	**<0.001**	0.207	**<0.001**	**<0.001**	0.103
Fractional shortening (%)	**<0.001**	**<0.001**	0.183	0.003	**<0.001**	0.114
LVO (mL/min/kg)	**0.001**	**<0.001**	0.076	**0.001**	**<0.001**	0.015
*Speckle tracking parameters*						
Global longitudinal peak systolic strain (%)	0.032	0.597	0.407	0.084	0.791	0.150
Global longitudinal peak systolic strain rate (1/sec)	**<0.001**	0.009	0.098	0.006	0.009	0.080
Longitudinal peak systolic strain (%)						
Septum						
basal	0.829	0.916	0.187	0.287	**<0.001**	0.118
mid	0.960	0.592	0.254	0.213	**<0.001**	0.037
apical	0.711	0.003	0.254	0.359	0.006	0.023
Left wall						
basal	0.097	0.960	0.042	0.111	**<0.001**	0.060
mid	0.159	0.218	0.020	0.107	0.334	0.062
apical	0.435	0.087	0.044	0.748	0.111	0.049
Longitudinal peak systolic strain rate (1/sec)						
Septum						
basal	0.214	0.260	0.048	0.241	0.241	0.407
mid	0.085	0.039	0.217	0.084	0.057	0.104
apical	0.016	0.016	0.696	0.184	0.018	0.304
Left wall						
basal	0.012	0.046	0.591	0.179	0.043	0.539
mid	**<0.001**	0.591	0.107	0.004	0.584	0.335
apical	0.112	0.214	0.371	0.071	0.279	0.483

Presented are the p-values of the ANOVA for repeated measurements with the patient group as between-subject factor; p-values for statistically significant effect after Bonferroni correction (p<0.0023) are printed in bold.

DOL - Day of life, LVO - Left ventricular output.

**Figure 2:**
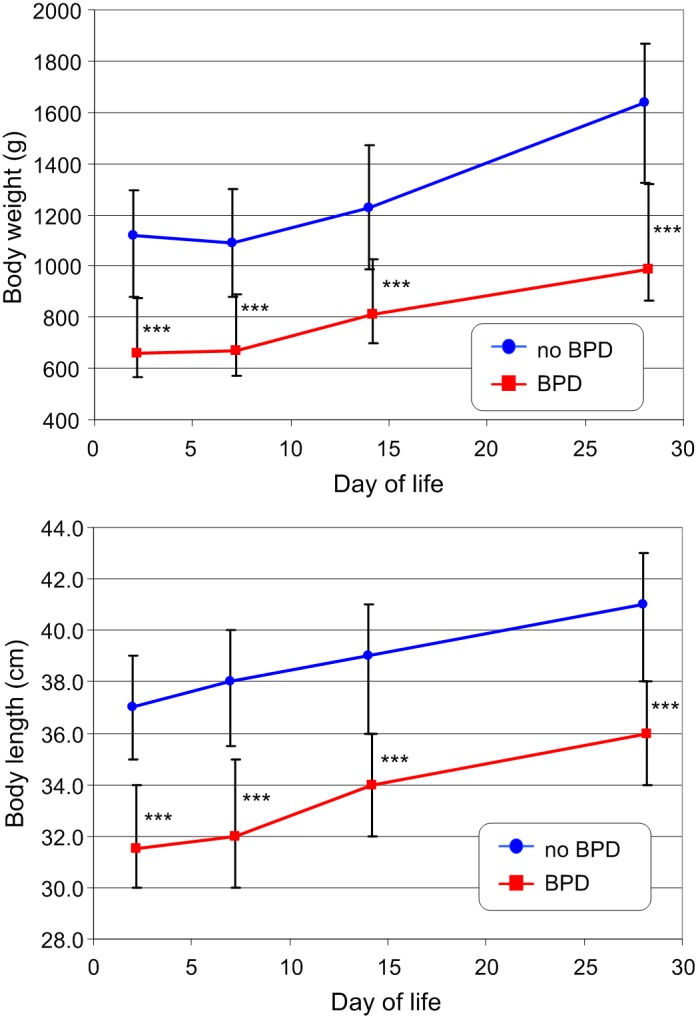
Development of body weight (top) and body length (bottom) in VLBW infants with and without BPD during the first four weeks of life. Presented are median with interquartile range; statistical significant differences between the patient groups are marked: *** - p<0.001.

**Figure 3:**
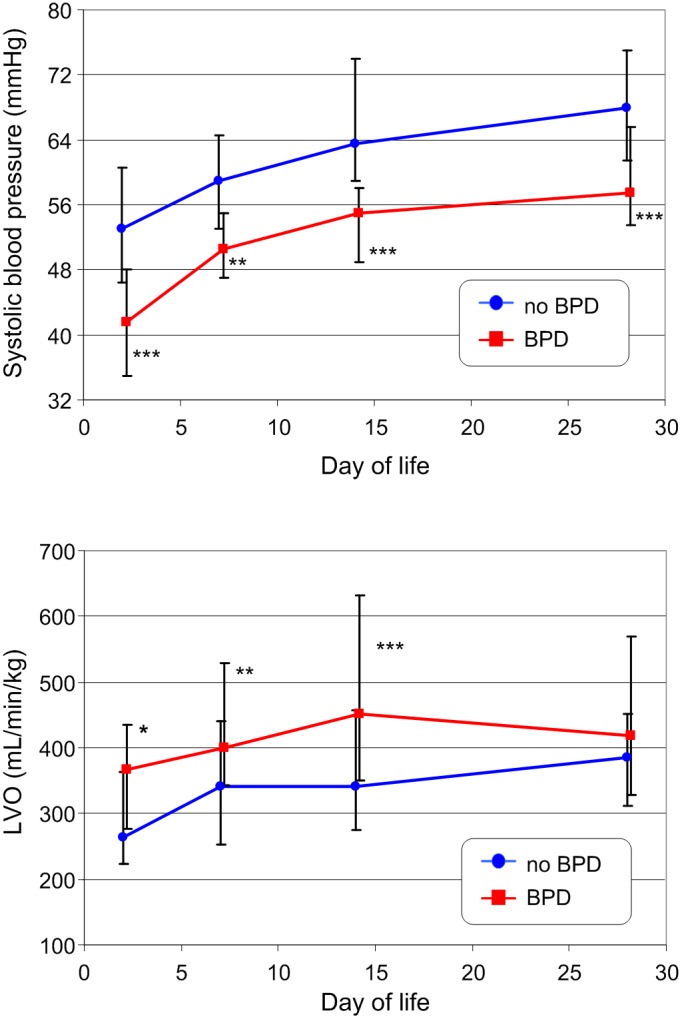
Development of systolic blood pressure (top) and left ventricular cardiac output (bottom) in VLBW infants with and without BPD during the first four weeks of life (the mode of presentation is the same as that in Figure 2). * - p<0.05; ** - p<0.01; *** - p<0.001

**Figure 4:**
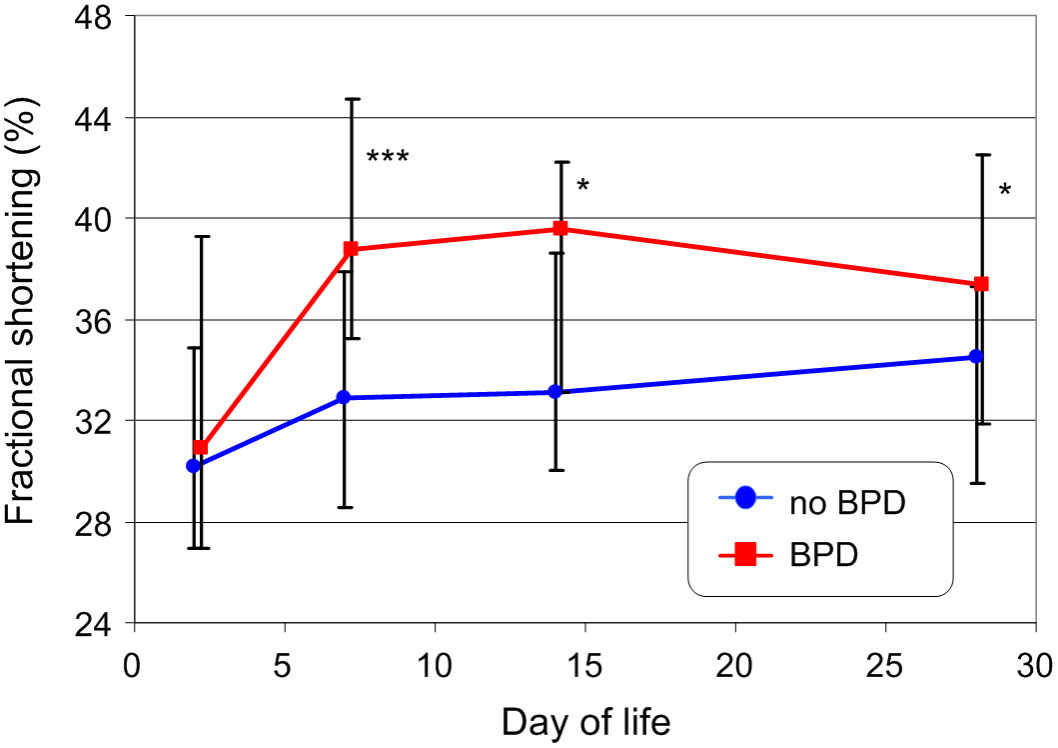
Development of fractional shortening in VLBW infants with and without BPD during the first four weeks of life (the mode of presentation is the same as that in Figure 2). * - p<0.05; *** - p<0.001

The development of bodyweight and body length in infants with and without BPD at day 28 is shown in Fig. 2. Despite the overall lower average weight and gestational age of infants with BPD, they evidenced comparable growth to infants without BPD. Similar development was seen for the blood pressure values as shown by systolic blood pressure exemplarily (Fig. 3). Parameter development of LVO (Fig. 3) and FS (Fig. 4) was more complex. Both were higher in patients who developed a BPD compared to infants without BPD. LVO differed statistically significantly between the two patient groups at day 7 and 14, though these differences disappeared by day 28. FS showed the greatest variation at day 7, after which the difference decreased. We did not find a significant correlation between both parameters and heart rate (all p-values>0.11) at any time of measurement.

Of the speckle tracking parameters, only the development of GLSSR and LSSR for the left wall mid segment were different between infants with and without BPD (both p<0.001). The effect of hsPDA on both parameters was lower with p = 0.006 for GLSSR and p = 0.004 for LSSR (Table 6) and after Bonferroni correction (p<0.0023) above the limit of statistical significance.

**Figure 5:**
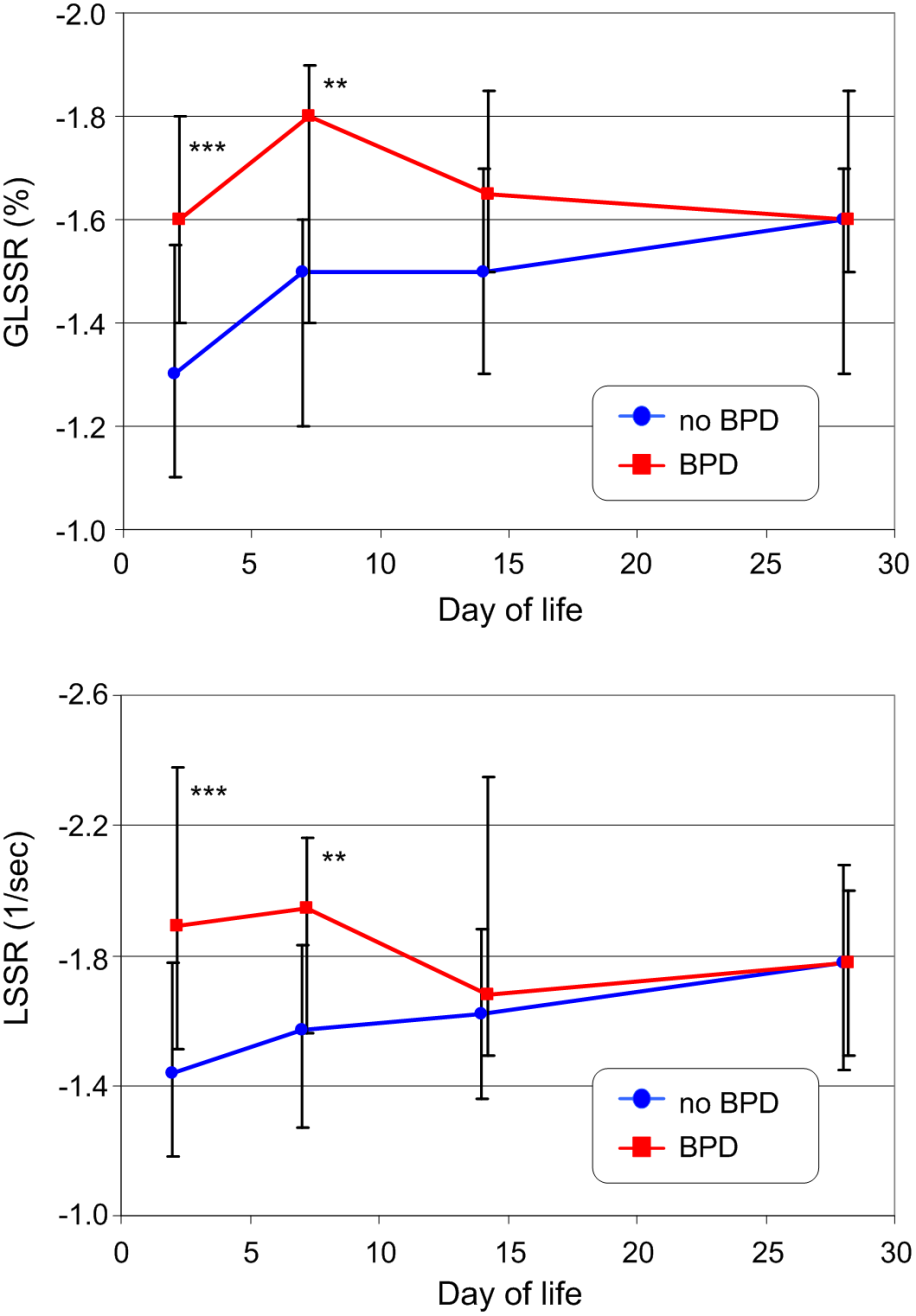
Development of the global longitudinal peak systolic strain rate (GLSSR) (top) and the longitudinal peak systolic strain rate (LSSR) for the mid left wall segment (bottom) in VLBW infants with and without BPD during the first four weeks of life (the mode of presentation is the same as that in Figure 2). ** - p<0.01; *** - p<0.001

The development of GLSSR and LSSR in infants with and without BPD at day 28 is shown in Fig. 5. In infants without BPD, there was a slight increase of the absolute GLSSR value during the first two weeks. In infants with BPD, values were significantly higher during the first week of life after which the differences disappeared. As with GLSSR, there was an increase in left wall mid segment LSSR during the first two weeks in infants without BPD, whereas in infants who developed a BPD the values were always higher and trended slightly down. The differences were greatest at day 1 and decreased during the first 4 weeks of life. At day of life 28, the left wall mid segment LSSR was nearly the same for infants with and without BPD. There was no statistically significant correlation between both speckle tracking parameters and heart rate except for GLSSR at day 1 (*Rs* = −0.2653, p = 0.015) which would disappear after Bonferroni correction.

## Discussion

This study has shown that speckle tracking measurements of the left ventricle can be performed in VLBW infants with good reproducibility for the longitudinal systolic strain and strain rate, especially of the mid septum. While anthropometric parameters show rapid development during these infants’ first 4 weeks of life [24], the speckle tracking parameters did not differ statistically significant during the neonatal period. To our knowledge, the present study is the first single-center study in which the development of speckle tracking parameters was investigated in infants who subsequently did and did not develop BPD.

In contrast to the development of the anthropometric parameters, our results demonstrate that initial age-related changes in LSS of the septum apical segment diminished after the second week of life. This agrees with the findings Seghal et al 2011 achieved using conventional hemodynamic measurements [25]. They described the rapid changes that occur in cardiovascular adaptation during the transition from intrauterine to extrauterine life for preterm birth. The elevation of the speckle tracking parameters in the first week of life is also associated with changes in some physiologically important aspects of neonatal hemodynamic and conventional echocardiographic parameters, including cardiac output, heart rate, blood pressure and FS as shown in Table 5.

Additionally, the myocardium in VLBW infants is exposed to additional significant changes in loading conditions during the early neonatal period. A PDA is a very common finding in preterm infants that results in chronic left ventricular volume loading. This left ventricular volume loading may be reflected in our results on the development of conventional hemodynamic and echocardiographic parameters, because most infants in the BPD group who have increased FS and LVO at day 7 and 14 (Fig. 3 and 4) needed a subsequent PDA intervention.

The changes in hemodynamic parameters associated with PDA have been well studied in surfactant-treated preterm lambs [26] and confirmed in preterm neonates using Doppler echocardiography [27]. Premature lambs were able to more than double their left ventricular output when challenged with increasing degrees of left-to-right shunt through a PDA. This increase in cardiac output was achieved through an increase in stroke volume, primarily as a result of decreased afterload resistance on the heart and increased left ventricular preload. Lindner et al. and Shimada et al. confirmed that under the hemodynamic conditions associated with symptomatic PDA, ventilated preterm neonates increased LVO by increasing stroke volume [28], [29]. This was also reflected in our results, where we did not find any correlation between LVO and heart rate. Thus we expected hsPDA would have a greater influence on the speckle tracking parameters than the development of a BPD, but surprisingly found only a statistically significant influence of BPD here. Unfortunately, we could not investigate the effect of hsPDA and BPD patients separately because most patients with BPD needed PDA intervention. Furthermore, although there are studies that associate PDA with the development of BPD, there is no literature that identifies specific clinical predictors of BPD in preterm infants. Moreover, in their study Chock et al. found that only lower gestational age, and not PDA treatment or Echo score, was associated with the adverse outcome of BPD [30].

Myocardial strain rates are strong indices of LV contractility [31], [32]. Thus far no studies have identified which strain rate changes derived from speckle tracking are detectable in preterm infants with and without BPD development. Our speckle tracking measurements revealed that infants with BPD have higher GLSSR than infants without these morbidities. These findings were also detectable in the strain rate measurement of the left wall mid segment. We speculate that this free wall segment of the LV is quite sensitive to changes in myocardial contractility.

Although recent studies have described not only alterations in right ventricular function in patients with BPD and pulmonary hypertension but also alterations in LV [21], [33]–[35], the mechanisms of these left ventricular alterations are not fully understood. In a previous paper from our group, we showed that the right ventricular index of myocardial performance values was significantly higher in infants with a subsequent diagnosis of BPD compared to controls, indicating increased pulmonary vascular resistance (PVR) [36]. But we did not measure pulmonary artery pressure and resistance because, to our knowledge, the sensitivity of the commonly used echocardiographic parameters (e.g. tricuspid regurgitant jet, septal displacement, or right atrial enlargement) is questionable in preterm infants and the invasive measurement of PVR is difficult to perform in neonates. Thus we were unable to demonstrate that the observed increment in the speckle tracking parameters in our patients who developed a BPD occurred as consequence of increased PVR. Moreover, our BPD patients did not have significant left ventricular hypertrophy or systemic hypertension.

The etiology of LV changes in children and adults with chronic lung disease is still poorly understood. The altered LV function in patients with obstructive pulmonary disease has been explained by either a shift of the interventricular septum toward the LV cavity or circumferential myocardial fiber changes that impact contractile function [37]. Our patients did not show evidence of septal flattening or a shift into the LV cavity, and we found no differences in the septal speckle tracking measurements.

Many studies have also shown that infants with severe BPD develop systemic hypertension, and that the abnormal LV myocardial performance index may be caused by changes in systemic vascular resistance [38], [39]. No patients in our study had systemic hypertension though. This might explain why we did not find addition changes in LV in our study group, as described by Hardegree et al. [21].

In our population, the magnitude of longitudinal deformation was different than in other studies that investigated speckle tracking parameters in mature newborns, infants or adolescents. Lorch et al. and Marcus et al. describe normal values and maturational changes in LV longitudinal deformation from birth to adolescence in large pediatric cohorts [9], [10]. The authors found strain values of approximately 18% for the age group below 1 year. Conversely, Elkiran et al. found lower values for the longitudinal deformation of the left ventricle in less immature newborns with a gestational age between 36 and 37 weeks. It is difficult to compare our results to these studies because they were performed using a variety of ultrasound equipment and speckle tracking analysis software. Moreover, we think that lower longitudinal deformation values in VLBW infants are most likely explained by the immaturity of the heart and the higher alterations in cardiac loading conditions, contractility, myocardial function and histological structure of the ventricular myocardium after birth.

This study has a number of strengths and limitations. The main strengths include the longitudinal measurements over 4 weeks using a large sample size with the same investigator. We used the same equipment and protocol for all patients. The study was limited by offline speckle tracking analysis. We were unable to investigate the effect of mechanical ventilation on parameter development in BPD infants because nearly all (94%) BPD infants required mechanical ventilation during the first days of life. Mechanical ventilation could alter the pre- and after-load conditions, though we found no such effect in a previous study [36]. Still we cannot exclude that due to the poor tracking in the apical region, n-CPAP or mechanical ventilation might have large impacts on the measurements. Furthermore, since the number of BPD infants was relatively low, we did not define the severity of BPD. Because almost all very immature infants developed a BPD, we cannot assess the extent to which prematurity per se contributes to the differences in parameter development. Thus we are unable at present further correlate changes in LV performance with the severity of BPD.

The Bonferroni correction used to counteract the problem of multiple comparisons is a common method. However, it is a conservative method and may increase type 2 errors. There are also technical limitations. The dependence of 2D-STE imaging on the frame-by-frame tracking of the myocardial pattern means it is influenced by image factors, including reverberation artefacts and attenuation. Finally, we measured only longitudinal strain and strain rate, and not radial or circumferential strain.

In conclusion, our study demonstrates that measuring LV longitudinal systolic strain and strain rate derived by speckle tracking is feasible in VLBW infants. The development of the derived parameters differs from the development of anthropometric and hemodynamic parameters during the first weeks of life and is also influenced by morbidity such as BPD. Further studies should focus on real-time speckle tracking and volume monitoring because this new technique allows LV volumes to be measured without manual tracing.
